# The antimicrobial resistance crisis needs action now

**DOI:** 10.1371/journal.pbio.3001918

**Published:** 2022-11-23

**Authors:** Nonia Pariente, on behalf of the PLOS Biology Staff Editors

**Affiliations:** Public Library of Science, San Francisco, California, United States of America and Cambridge, United Kingdom

## Abstract

Antimicrobial resistance is a global problem of increasing proportions that we cannot afford to look away from. Our Editorial shines a light on the crisis and ways we can all help to address it.

## Teaser

Antimicrobial resistance is a global problem of increasing proportions that we cannot afford to look away from. This World Antimicrobial Awareness Week, we shine a light on the crisis and ways we can all help to address it.

Can you imagine a world in which your life could be at serious risk from a superficial wound or from something you ate because there were no drugs to treat you? This scenario seems dystopic but is within the realm of possibility, and already occurs with certain infections. Antimicrobial resistance (AMR) is considered by the World Health Organization (WHO) to be one of the top 10 global public health threats.

As we end year three of the COVID-19 pandemic, our response to the AMR crisis—already insufficient before the pandemic—needs urgent action, coordination and funding. The reallocation of resources, overwhelmed hospitals and doctors, and over-prescription of antibiotics during these years have set us back. According to the Centers for Disease Control and Prevention, in the United States alone AMR increased by 15% between 2019 and 2020 [1]. Years of progress are estimated to have been lost in the global fight against tuberculosis, including the first increase in drug-resistant infections in many years [2]. There has also been a substantial increase in sexually transmitted infectious diseases, many of which are resistant to antimicrobials; in 2020 there were 83 million new cases of multi-drug-resistant gonorrhea worldwide. World Antimicrobial Awareness Week is a global campaign observed annually on 18–24 November to promote awareness and understanding of AMR and to inform of best practices to reduce its emergence and spread. This year, we are participating in this effort to bring some needed attention to the AMR crisis and how it can be tackled, including the basic and applied research that *PLOS Biology* would be happy to give a voice to.

In a Perspective in this issue [3], Dolecek and colleagues discuss the GRAM report, published earlier this year [4], which estimated that bacterial AMR contributed to over 4 million deaths and was directly responsible for over 1.2 million deaths in 2019. As they say, “the momentum and urgency to tackle AMR has faded into the background—yet the AMR crisis continues to worsen” [3]. Their article discusses the global inequalities in the burden of AMR and steps that should be taken to stem infection spread, especially in the most vulnerable countries.

Even further from the spotlight are fungal and parasite AMR, despite being a threat to both public health and food security. Resistance to antifungals can occur in previously susceptible fungi, but there are also new emerging species, such as the yeast *Candida auris*, that are already resistant to available antifungals [5]. With few exceptions, fungal and parasite infections receive little attention and resources, to the extent that there’s insufficient high-quality data on disease distribution and resistance to even estimate their exact burden, never mind fund the development of new diagnostic tools or antimicrobials. Recognizing this neglect, the WHO has just published a fungal priority pathogens list [6]—in the footsteps of their 2017 list of bacterial pathogens—to guide research, development and public health action.

Although it is a global problem, low- and middle-income countries bear a disproportionate amount of the burden of AMR, irrespective of pathogen. The maxim oft repeated during the COVID-19 pandemic “nobody is safe until we are all safe” also applies here—it is a global responsibility to help all countries deal with the burden of AMR. As Dolecek et al. discuss [3], infection prevention and control are unmet objectives in low- and middle-income settings, where basic sanitation measures are glaringly lacking. That interventions that require no technological or pharmacological development and are cheap to implement, such as access to clean water and other basic hygiene measures, are still lacking in a large fraction of the world is frankly unacceptable and should galvanize us to action. This is something that can and should be addressed with urgency, before we run out of medical options to treat drug-resistant infections.

The WHO developed a roadmap to address AMR that was internationally ratified at the 2015 World Health Assembly [7]. This plan calls for reducing the number of infections, but also for better surveillance, optimal use of existing antibiotics and the development of novel drugs, vaccines and diagnostic tests. Good antimicrobial stewardship in both human and animal settings is necessary to prevent the selection and further spread of resistance. It will be important not to step backwards in countries with good oversight by introducing policies such as one recently discussed in the United Kingdom, in which antibiotic prescription would be devolved to pharmacists to shorten doctor waiting lists. While the aim was good, the means might prove disastrous.

AMR surveillance is crucial to detect the emergence and spread of resistance in different ecological settings and could, if combined with phenotypic assays, point to new resistance mechanisms (we have recently published research along these lines [8,9,10]). We can learn lessons on surveillance from the COVID-19 pandemic response and should capitalize on the infrastructure that was developed to track SARS-CoV-2 by providing stable funding to maintain and increase global genomic capacitation, strengthen surveillance networks that can pivot to multiple pathogens, and maintain the rapid sharing of genomic data. The WHO has just published a set of principles for pathogen genome data sharing [11], calling for global capacity development, collaboration and cooperation for sampling, sequencing and data analysis, sharing the data as early and as openly as possible, and the transparent acknowledgement of intellectual credit. We would do well to heed this advice.

Development of rapid diagnostic tests, especially those that can be easily deployed in resource-limited settings, and renewed drug discovery efforts are also pillars of the response to the AMR crisis and topics that *PLOS Biology* has and will continue to cover [12,13]. Developing drugs to be stored and used sparingly as a last resort is financially difficult for the pharmaceutical industry; to address this, several public–private partnerships for antimicrobial development have been established in the past few years, such as CARB-X and GARDP. In one such partnership, GlaxoSmithKline (GSK) and the US Government BARDA office have reported that a new, first-in-class antibiotic, gepotidacin, has been so successful in a phase III clinical trial against urinary tract infections that the trial was halted and GSK will soon file for drug approval. They are also studying efficacy against gonorrhea in another phase III study. Because gepotidacin has a new mechanism of action, it could be effective against drug-resistant bacteria. These encouraging results showcase the potential of these partnerships to drive antimicrobial drug discovery.

In addition to drugs, investment and research should be geared towards finding vaccines, looking beyond viruses to include those against bacteria, fungi and parasites. The resounding success of the mRNA vaccines against COVID-19 has given new impetus to the possibility of using new technology to find effective vaccines against pathogens for which slow progress had previously thwarted vaccine development.

The pandemic threw us off track, and the current world situation with war in Ukraine, drought and famine in large regions of Africa, flooding and other devastating effects of climate change, as well as a global economic recession, pulls our collective attention in many directions. However, we cannot ignore the pressing need to address the AMR crisis. Let us use this World Antimicrobial Awareness Week to gain momentum and discuss ways we can collectively tackle AMR.
